# Comprehensive analysis of the 21-gene recurrence score in invasive ductal breast carcinoma with or without ductal carcinoma in situ component

**DOI:** 10.1038/s41416-020-01212-w

**Published:** 2020-12-17

**Authors:** Yufei Zeng, Weiqi Gao, Xiaosong Chen, Kunwei Shen

**Affiliations:** Department of General Surgery, Comprehensive Breast Health Center, Ruijin Hospital, Shanghai Jiao Tong University School of Medicine, Shanghai, China

**Keywords:** Genetics research, Surgical oncology, Breast cancer, Breast cancer

## Abstract

**Background:**

Invasive ductal carcinoma (IDC) is often accompanied by ductal carcinoma in situ (DCIS). Whether the DCIS component affects the 21-gene recurrence score (RS) is unclear.

**Methods:**

Consecutive ER-positive, HER2-negative, N0–1 patients with RS results were included. Patients were divided into pure IDC and IDC with DCIS (IDC/DCIS) groups. The RS, the expression of its 16 cancer genes and prognosis were compared between IDC and IDC/DCIS patients.

**Results:**

A total of 1458 patients were enrolled, 320 of whom had concomitant DCIS. DCIS component was independently associated with lower RS (*P* = 0.038). IDC/DCIS patients more often had a low-risk RS (*P* = 0.018) or intermediate-risk RS (*P* = 0.024). Regarding individual genes in the RS panel, Ki67, CCNB1 and MYBL2 in the proliferation group and MMP11 and CTSL2 in the invasion group were significantly lower among IDC/DCIS patients than pure IDC patients. Among IDC/DCIS patients, lower RS was independently correlated with a higher DCIS proportion and lower DCIS grade. Within a median follow-up of 31 months, the DCIS component in IDC did not significantly influence prognosis.

**Conclusions:**

IDC with DCIS component is associated with a lower 21-gene RS, possibly due to lower expression of proliferation and invasion genes. DCIS proportion and grade independently influenced the 21-gene RS in IDC/DCIS patients. Due to the relatively short follow-up period and low recurrence rate, the impact of the DCIS component in IDC on prognosis needs further evaluation.

## Background

Ductal carcinoma in situ (DCIS) is a non-invasive disease of the breast that is treated with different strategies. Although DCIS may not be immediately life-threatening, it is suggested that DCIS is a precursor lesion to most, if not all, invasive breast carcinomas.^1,2^ It has been reported that 20.6–45.5% of IDC tumours have an accompanying DCIS component (IDC/DCIS).^3–6^ However, whether IDC/DCIS possesses the same biological aggressiveness as pure IDC remains undefined. Previous studies have demonstrated that the DCIS component in IDC is correlated with lower proliferation and metastatic potential, and is associated with a lower risk of local recurrence, especially if the ratio of DCIS to IDC size is high.^7,8^

The 21-gene recurrence score (RS) assay has been widely used to assess the risk of disease recurrence among early-stage invasive breast cancer patients.^9–12^ Both the American Society of Clinical Oncology (ASCO) and National Comprehensive Cancer Network (NCCN) guidelines have recommended the 21-gene RS in the management of oestrogen receptor (ER)-positive, human epidermal growth factor receptor 2 (HER2)-negative early-stage invasive breast cancer patients. Since the 21-gene assay genetically assesses the proliferative and invasive propensity of the tumour, it would be reasonable to assume that the DCIS component may also affect the 21-gene assay result. However, whether and how the DCIS component in IDC affects 21-gene RS assay testing remains unknown.

Based on the above questions, this study aims to explore whether and how the DCIS component in IDC impacts the 21-gene RS by analysing individual gene expression between IDC and IDC/DCIS groups. Moreover, we will also analyse factors associated with 21-gene RS among IDC/DCIS patients and whether the DCIS component influences adjuvant chemotherapy usage and prognosis.

## Methods

### Patients and materials

Consecutive patients who were diagnosed with IDC and treated in Comprehensive Breast Health Center, Ruijin Hospital, from January 2009 to December 2018, were retrospectively included. The inclusion criteria of the study were as follows: (1) female, (2) histologically proven ER-positive, HER2-negative invasive breast cancer, (3) previous mastectomy or lumpectomy with negative margins, (4) axillary lymph node negativity or no more than 3 positive lymph nodes and (5) a 21-gene RS test. The exclusion criteria were as follows: (1) pT4 disease, (2) presence of any invasive cancer component other than IDC, (3) bilateral breast cancer, (4) metastatic breast cancer, (5) previous or concurrent malignant disease or (6) previous neoadjuvant systemic therapy. Clinicopathological information was obtained from the Shanghai Jiao Tong University Breast Cancer Database (SJTU-BCDB), including age, surgery of the breast, tumour stage, nodal status, pathology, grade, ER expression, progesterone receptor (PR) expression, Ki67 expression and 21-gene RS results. Information regarding adjuvant treatment and disease outcome was also retrieved from the SJTU-BCDB. The follow-up information was based on all data available before 31 December 2019. The current study was reviewed and approved by independent ethics committees of Ruijin Hospital, and the research met the requirements for the protection of patients.

### Study design

All enrolled patients were ER and/or PR positive. The cut-off point of high ER expression was set at 50%, and the cut-off point of high PR expression was set at 20% according to the St Gallen consensus.^13,14^ Moreover, high Ki67 expression was set at 14%. Luminal A breast cancer was defined as ER positive, HER2 negative with PR expression ≥20% and Ki67 < 14%, while luminal B breast cancer was defined as ER positive, HER2 negative with PR expression <20%, or Ki67 ≥ 14%.^14^ According to histological type, patients were divided into the pure IDC group and the IDC accompanying DCIS component (IDC/DCIS) group. Among the IDC/DCIS group, patients were further divided into two groups according to the proportion of DCIS components: the IDC/DCIS < 50% group for tumours containing DCIS smaller than the IDC component and the IDC/DCIS ≥ 50% group for tumours containing DCIS equal to or larger than the IDC component.

### Analysis of 21-gene RS

The 21-gene RS assay was performed locally at our centre. Detailed information on the 21-gene RS analysis was presented in our previous work.^15^ In brief, macrodissection was performed to ensure that tumour elements accounted for more than 50% of the tissue. Subsequently, RNA was extracted from three 10-μm unstained sections of formalin-fixed, paraffin-embedded (FFPE) tissue using the RNeasy FFPE RNA kit (Qiagen, 73504, Germany). Gene-specific reverse transcription was performed using an Omniscript RT kit (Qiagen, 205111, Germany). Quantitative reverse transcriptase polymerase chain reaction (RT-PCR) was done using Premix Ex Taq^TM^ (TaKaRa Bio, RR390A) in an Applied Biosystems 7500 Real-Time PCR System (Foster City, CA). In this assay, the expression of 16 cancer genes is measured in triplicate and normalised to a set of 5 reference genes. The RS result, ranging from 0 to 100, was derived from the reference-normalised expression of the 16 cancer genes. According to the 21-gene RS results, patients were categorised into low-risk (RS < 18), intermediate-risk (RS 18–30) and high-risk (RS ≥ 31) groups.^9,10,12^ For further analysis, the individual gene expression of the 16 cancer genes was measured, and the distribution of the 16-cancer gene expression in IDC and IDC/DCIS patients was analysed.

### Statistical analysis

All statistical analyses were carried out by SPSS version 18.0 (SPSS, Inc., Chicago, IL). Chi-square tests and logistic regression analysis were used to assess the distribution of characteristics in different subgroups. All missing values are missing at complete random; therefore, pairwise deletion was performed when conducting the chi-square test. The Mann–Whitney test or Kruskal–Wallis test was used to assess the distribution of the 21-gene RS as a continuous variable in the different subgroups, and to compare the expression levels of the 16 cancer genes between subgroups. Survival analysis was performed by Kaplan–Meier estimates and log-rank tests, and the distant recurrence-free interval (DRFi) was used as the primary prognostic endpoint. DRFi was defined as the time from surgery to distant recurrence or death from breast cancer.^16^ All statistical tests were 2 tailed, and *P* < 0.05 was considered significant.

## Results

### Patient and tumour characteristics

Of the 2894 female patients with ER-positive, HER2-negative, IDC breast cancer eligible for 21-gene testing, 1758 (60.7%) eventually underwent the 21-gene RS assay. A total of 1458 patients were enrolled in our study (Supplementary Fig. 1). Detailed patient and tumour characteristics are listed in Table 1. At the time of diagnosis, 978 patients (67.1%) were over 50 years old. The study comprised 771 patients (52.9%) receiving mastectomy and 687 patients (47.1%) receiving lumpectomy. T1 tumours were present in 1039 patients (71.3%) and T2–3 in 414 patients (28.4%). Node negativity was found in 1224 patients (84.9%), and 234 patients (16.0%) had positive nodes. There were 141 patients (9.7%) diagnosed with IDC grade I tumours, whereas 965 patients (66.2%) and 336 patients (23.0%) were diagnosed with IDC grade II and III tumours, respectively. ER expression ≥50% was found in 1,355 patients (92.9%), while PR expression ≥20% was found in 1006 patients (69.0%). The luminal A and B subtypes accounted for 30.7% and 69.3% of the study population, respectively. Regarding the 21-gene RS results, 291 patients (20.0%) were categorised into the low-RS group, while 762 (52.3%) and 405 (27.8%) patients were categorised into the intermediate- and high-RS groups, respectively.

**Table 1:** Patient and tumour characteristics in the whole population and in different subgroups.

Characteristics	Total, no. (%)	Histological type		*P*
		Pure IDC, no. (%)	IDC/DCIS, no. (%)	
Total	1458 (100.0%)	1138 (78.1%)	320 (21.9%)	
*Age (years)*				**<0.001**
<50	480 (32.9%)	346 (30.4%)	134 (41.9%)	
≥50	978 (67.1%)	792 (69.6%)	186 (58.1%)	
*Surgery type*				**0.008**
Mastectomy	771 (52.9%)	581 (51.1%)	190 (59.4%)	
Lumpectomy	687 (47.1%)	557 (48.9%)	130 (40.6%)	
*Tumour stage*^a^				0.273
T1	1039 (71.3%)	818 (72.2%)	221 (69.1%)	
T2–3	414 (28.4%)	315 (27.8%)	99 (30.9%)	
*Nodal status*				0.649
pN0	1224 (84.0%)	958 (84.2%)	266 (83.1%)	
pN1	234 (16.0%)	180 (15.8%)	54 (16.9%)	
*IDC grade*^b^				**<0.001**
I	141 (9.7%)	93 (8.3%)	48 (15.2%)	
II	965 (66.2%)	754 (67.0%)	211 (66.8%)	
III	336 (23.0%)	279 (24.8%)	57 (18.0%)	
*Molecular subtype*				**0.001**
Luminal A	448 (30.7%)	326 (28.6%)	122 (38.1%)	
Luminal B	1010 (69.3%)	812 (71.4%)	198 (61.9%)	
*ER expression*				**0.034**
<50%	103 (7.1%)	89 (7.8%)	14 (4.4%)	
≥50%	1355 (92.9%)	1049 (92.2%)	306 (95.6%)	
*PR expression*				**0.019**
<20%	452 (31.0%)	370 (32.5%)	82 (25.6%)	
≥20%	1006 (69.0%)	768 (67.5%)	238 (74.4%)	
*Ki67 expression*^c^				0.100
<14%	647 (44.4%)	492 (43.3%)	155 (48.4%)	
≥14%	810 (55.6%)	645 (56.7%)	165 (51.6%)	
RS (mean)	26.3	27.0	23.9	**<0.001**
*RS category*				**0.002**
Low	291 (20.0%)	217 (19.1%)	74 (23.1%)	
Intermediate	762 (52.3%)	580 (51.0%)	182 (56.9%)	
High	405 (27.8%)	341 (30.0%)	64 (20.0%)	

*IDC* invasive ductal carcinoma, *DCIS* ductal carcinoma in situ, *ER* oestrogen receptor, *PR* progesterone receptor, *RS* recurrence score.

Significant P-value (<0.05) are in bold.

^a^Tumour size unknown in five patients.

^b^IDC grade unknown in 16 patients.

^c^Ki67 unknown in one patient. All missing values are missing at complete random; therefore, pairwise deletion was performed when conducting chi-square test.

### Clinicopathological factors and 21-gene RS difference between the IDC and IDC/DCIS groups

**Fig. 1:**
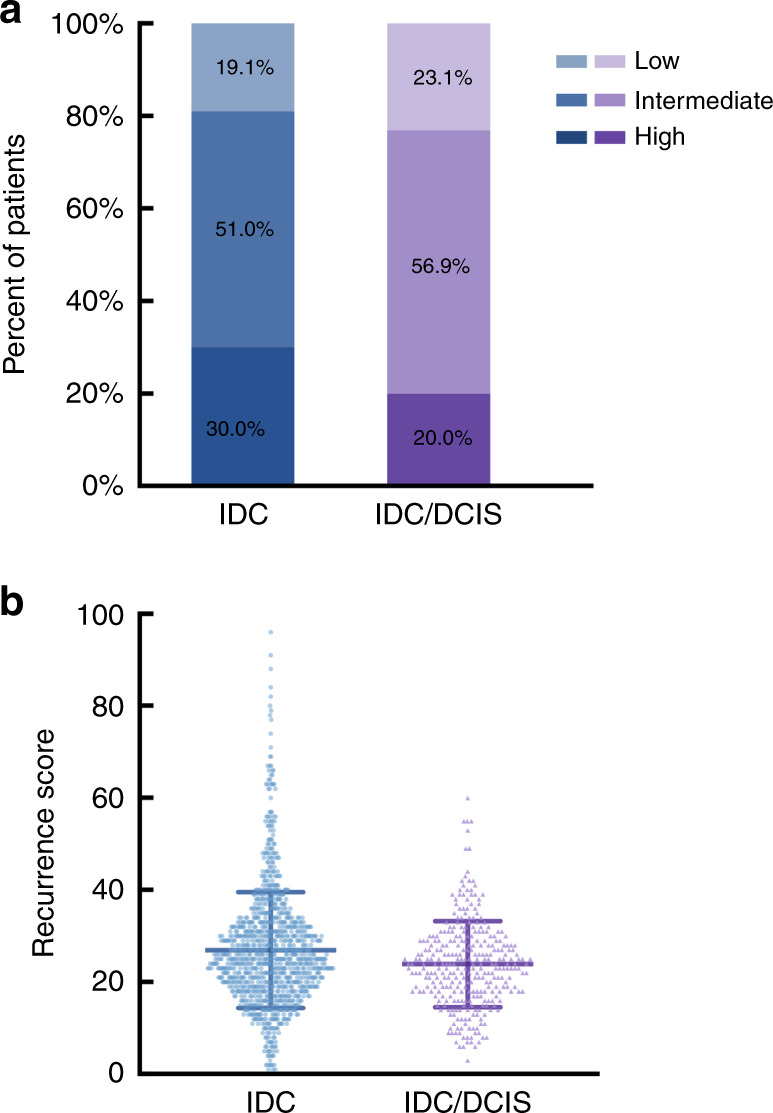
Distribution of the 21-gene RS in breast cancer patients with IDC or IDC/DCIS. **a** In all, 21-gene RS as a categorical variable (chi-square test *P* = 0.002); **b** 21-gene RS as a continuous variable (Mann–Whitney test *P* < 0.001).

Among all patients, 1138 patients (78.1%) had pure IDC tumours, and 320 patients (21.9%) had DCIS components. In univariate analysis, concomitant DCIS in IDC was significantly associated with age (*P* < 0.001), surgery type (*P* = 0.008), IDC grade (*P* < 0.001), molecular subtype (*P* = 0.001), ER expression (*P* = 0.034), PR expression (*P* = 0.019) and 21-gene RS (*P* = 0.002) (Table 1). The proportions of low-, intermediate- and high-risk RS were 19.1%, 51.0% and 30.0%, respectively, among patients with pure IDC, and 23.1%, 56.9% and 20.0% in the IDC/DCIS group (Fig. 1a, *P* = 0.002). The mean 21-gene RS in the pure IDC and IDC/DCIS groups was 27.0 and 23.9, respectively (*P* < 0.001, Fig. 1b). The histograms of the distribution of the 21-gene RS in different histological-type subgroups are presented in Supplementary Fig. 2. In multivariate analysis (Table 2), age (*P* < 0.001), IDC grade (*P* = 0.030) and 21-gene RS (*P* = 0.038) were significantly associated with the DCIS component in IDC patients. Compared with patients in the pure IDC group, IDC/DCIS patients more often had a low-risk RS (OR 1.62, 95% CI 1.09–2.42, *P* = 0.018) or an intermediate-risk RS (OR 1.47, 95% CI 1.05–2.05, *P* = 0.024).

**Table 2:** Multivariate analysis of different characteristics in the IDC/DCIS group compared to pure IDC group.

Characteristics	OR	95% CI	*P*
Age < 50 vs. ≥50	1.65	1.27–2.15	**<0.001**
*IDC grade*			**0.030**
I vs. III	1.89	1.15–3.10	**0.012**
II vs. III	1.17	0.83–1.64	0.378
Luminal A vs. B	1.25	0.91–1.72	0.166
ER < 50% vs. ≥50%	0.68	0.37–1.24	0.205
PR < 20% vs. ≥20%	1.07	0.77–1.51	0.682
*RS*			**0.038**
Low vs. high	1.62	1.09–2.42	**0.018**
Intermediate vs. high	1.47	1.05–2.05	**0.024**

*OR* odds ratio, *CI* confidence interval, *IDC* invasive ductal carcinoma, *DCIS* ductal carcinoma in situ, *ER* oestrogen receptor, *PR* progesterone receptor, *RS* recurrence score.

Significant P-value (<0.05) are in bold.

### Individual gene expression differences between IDC and IDC/DCIS patients

**Fig. 2:**
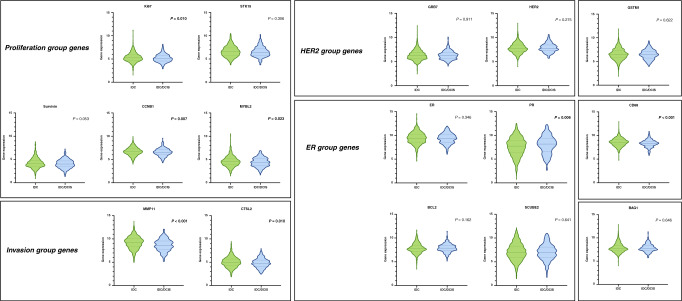
Individual gene expression levels of the 16 cancer genes from the 21-gene RS in breast cancer patients with IDC or IDC/DCIS. Genes are grouped on the basis of gene function and correlated expression. Proliferation group genes include Ki67, STK15, Survivn, CCNB1, and MYBL2. Invasion group genes include MMP11 and CTSL2. HER2 group genes include GRB7 and HER2. ER Proliferation group genes include ER, PR, BCL2, and SCUBE2. Significant P-value (<0.05) are in bold.

We further analysed the individual gene expression levels of the 16 cancer genes from the 21-gene RS. In total, there were seven genes that showed different expression between the IDC and IDC/DCIS groups, among which one gene was higher and six genes were lower in the IDC/DCIS group. The overall pattern observed was a decrease in the expression of proliferation and invasion genes in the the IDC/DCIS group compared to the IDC group (Fig. 2). The expression of Ki67 (*P* = 0.010), CCNB1 (*P* = 0.007) and MYBL2 (*P* = 0.023) in the proliferation group and the expression of MMP11 (*P* < 0.001) and CTSL2 (*P* = 0.010) in the invasion group were all significantly lower in the IDC/DCIS group than in the IDC group. The expression of genes from the HER2 and ER group did not differ significantly between the IDC and IDC/DCIS groups, except that the expression of PR was higher in the IDC/DCIS group than the IDC group (*P* = 0.006). Furthermore, the expression of CD68 was higher in the IDC group (*P* < 0.001), while the expression of GSTM1 and BAG1 did not differ significantly between groups.

### Factors impacting 21-gene RS among IDC/DCIS patients

**Fig. 3:**
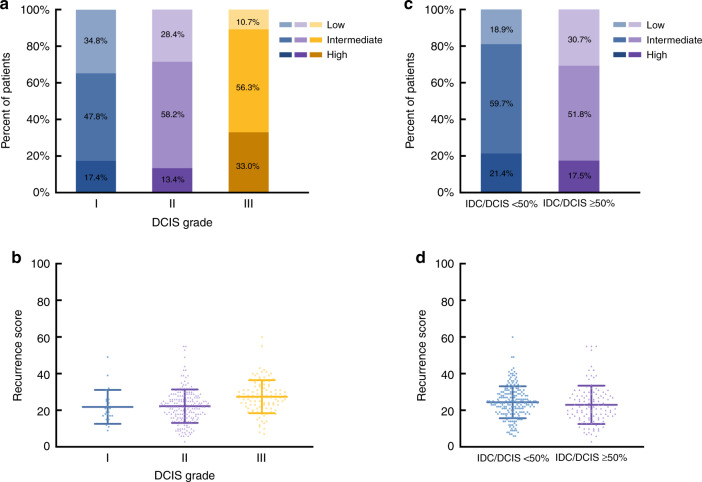
Distribution of 21-gene RS among IDC/DCIS patients. **a** In all, 21-gene RS as a categorical variable in patients with different DCIS grades (chi-square test *P* < 0.001); **b** 21-gene RS as a continuous variable in patients with different DCIS grades (Kruskal–Wallis test *P* < 0.001); **c** 21-gene RS as a categorical variable in patients with different proportions of DCIS (chi-square test *P* = 0.057); **d** 21-gene RS as a continuous variable in patients with different proportions of DCIS (Mann–Whitney test *P* = 0.064).

Among the 320 patients with IDC/DCIS, 206 patients (64.4%) had IDC/DCIS < 50% tumours, and 114 patients (35.6%) had IDC/DCIS ≥ 50% tumours. Regarding DCIS grade, 23 patients (7.2%), 194 patients (60.6%) and 103 patients (32.2%) had DCIS grade I, grade II and grade III tumours, respectively (Supplementary Table 1). Among IDC/DCIS patients, the 21-gene RS was significantly associated with DCIS grade (*P* < 0.001) and molecular subtype (*P* < 0.001) in univariate analysis. The proportions of low-, intermediate- and high-risk RS were 34.8%, 47.8% and 17.4% in the DCIS grade I group, 28.4%, 58.2% and 13.4% in the DCIS grade II group and 10.7%, 56.3% 33.0% in the DCIS grade III group (*P* < 0.001, Fig. 3a). The mean RS in the DCIS grade I, II and III groups was 21.9, 22.3 and 27.4, respectively (*P* < 0.001, Fig. 3b). The DCIS proportion was associated with the 21-gene RS with a nonsignificant trend in univariate analysis (*P* = 0.057). A total of 18.9%, 59.7% and 21.4% of patients with IDC/DCIS < 50% and 30.7%, 51.8% and 17.5% of patients with IDC/DCIS ≥ 50% had low-, intermediate- and high-risk RS (Fig. 3c). The mean 21-gene RS in the IDC/DCIS < 50% and IDC/DCIS ≥ 50% groups was 24.4 and 23.0, respectively (*P* = 0.064, Fig. 3d). Multivariate analysis showed that DCIS proportion (*P* = 0.022), DCIS grade (*P* = 0.001) and molecular subtype (*P* = 0.006) were all independent factors of the 21-gene RS in IDC/DCIS patients (Table 3). A low-risk RS (vs. high-risk RS, OR 2.53, 95% CI 1.18–5.43, *P* = 0.017) was more frequently present in IDC/DCIS ≥ 50% patients. Moreover, compared to DCIS grade III tumours, DCIS grade II tumours were correlated with a lower RS (low- vs. high-risk RS, OR 5.85, 95% CI 2.43–14.12, *P* < 0.001; intermediate- vs. high-risk RS, OR 2.25, 95% CI 1.20–4.24, *P* = 0.012).

**Table 3:** Multivariate analysis of different characteristics among IDC/DCIS patients with different 21-gene RS.

Characteristics	Low-risk RS*			Intermediate-risk RS*			*P*
	OR	95% CI	*P*	OR	95% CI	*P*	
DCIS proportion ≥ 50% vs. <50%	2.53	1.18–5.43	**0.017**	1.20	0.63–2.29	0.571	**0.022**
*DCIS grade*							**0.001**
I vs. III	4.13	0.95–17.89	0.058	1.20	0.34–4.27	0.779	
II vs. III	5.85	2.43–14.12	**<0.001**	2.25	1.20–4.24	**0.012**	
Luminal A vs. B	3.70	1.63–8.43	**0.002**	2.16	1.04–4.49	**0.039**	**0.006**

*OR* odds ratio, *CI* confidence interval, *IDC* invasive ductal carcinoma, *DCIS* ductal carcinoma in situ, *ER* oestrogen receptor, *PR* progesterone receptor, *RS* recurrence score.

*Reference category was high-risk RS.

Significant P-value (<0.05) are in bold.

### Individual gene expression differences among patients with different DCIS proportions

We subsequently analysed the expression of the 16 cancer genes between the IDC/DCIS < 50% and IDC/DCIS ≥ 50% groups. Among the genes whose expression differed significantly between the IDC and IDC/DCIS groups, only the expression of MMP11 and CD68 remained significantly different between the IDC/DCIS < 50% and IDC/DCIS ≥ 50% groups, while the expression of other proliferation or invasion genes was not different between the two groups (Supplementary Fig. 3). The expression of MMP11 and CD68 was significantly lower in the IDC/DCIS ≥ 50% group than in the IDC/DCIS < 50% group (MMP11 *P* < 0.001; CD68 *P* = 0.044). Different gene expression levels were also observed in GRB7 (*P* = 0.001), HER2 (*P* = 0.001), BCL2 (*P* = 0.006), SCUBE (*P* = 0.030) and BAG1 (*P* = 0.008), although the expression of these genes was not different between the IDC and IDC/DCIS groups.

### Chemotherapy recommendation and prognosis

Clinicopathological factors associated with chemotherapy administration are summarised in Supplementary Table 2. In total, 822 patients (56.4%) were recommended to receive chemotherapy. No significant difference in chemotherapy administration was observed between the pure IDC and IDC/DCIS groups (57.5% vs. 52.5%, *P* = 0.113). In univariate analysis, age, tumour size, nodal status, IDC grade, molecular subtype, ER expression, PR expression, Ki67 and 21-gene RS were significantly correlated with chemotherapy use (all *P* < 0.05, Supplementary Table 2). Further multivariate analysis showed that younger age (*P* < 0.001), larger tumour (*P* = 0.010), positive node (*P* < 0.001), higher IDC grade (*P* < 0.001), lower PR expression (*P* < 0.001), higher Ki67 (*P* < 0.001) and higher 21-gene RS (*P* < 0.001) were independent predictive factors for chemotherapy recommendation (Supplementary Table 3). Among low-risk RS patients, IDC patients were significantly more likely to be recommended with chemotherapy than IDC/DCIS patients (*P* = 0.030); however, no difference in chemotherapy recommendation between the two groups was observed in intermediate-risk (*P* = 0.773) or high-risk RS patients (*P* = 0.422, Supplementary Table 4).

The median follow-up time was 36 months, and a total of 25 DRFi events were observed. Details of the events are presented in Supplementary Table 5. No difference in DRFi was observed between pure IDC and IDC/DCIS patients (log-rank test *P* = 0.872, Supplementary Fig. 4). The 21-gene RS was significantly associated with DRFi among the entire patient cohort (log-rank test *P* = 0.049, Supplementary Fig. 2A) and was correlated with DRFi among pure IDC patients with borderline significance (log-rank test *P* = 0.081, Supplementary Fig. 5B). However, the 21-gene RS was not found to be significantly associated with DRFi among IDC/DCIS patients (log-rank test *P* = 0.305, Supplementary Fig. 5C), IDC/DCIS < 50% patients (log-rank test *P* = 0.558, Supplementary Fig. 6A) or IDC/DCIS ≥ 50% patients (log-rank test *P* = 0.510, Supplementary Fig. 6B). Similar results were also observed when analysing the DRFi event rate among RS risk groups: among all patients (chi-square test *P* = 0.010) and pure IDC patients (chi-square test *P* = 0.018), the rate of DRFi events was significantly different in different RS risk groups, but RS was no longer correlated with DRFi in IDC/DCIS patients (chi-square test *P* = 0.293, Supplementary Table 6). Univariate and multivariate Cox regression models are shown in Supplementary Tables 7 and 8, where only tumour stage (*P* = 0.009) was an independent prognostic factor among pure IDC patients.

## Discussion

The impact of the DCIS component in IDC on the 21-gene RS was undetermined. To our knowledge, the current study is the first to explore this issue. We found that the DCIS component in IDC is associated with a lower 21-gene RS, especially when the proportion of DCIS is high, and the DCIS grade is low. When analysing the expression of the 16 cancer genes from the 21-gene RS assay, we observed a significantly lower expression of proliferation and invasion genes among patients with concomitant DCIS.

While a substantial proportion of IDC tumours have concomitant DCIS, the influence of the DCIS component on tumour biological behaviour has been noticed. A study including 1355 patients with all molecular subtypes found that IDC grade and Ki67 level were significantly lower in patients with DCIS components in IDC disease.^8^ Another study conducted in ER-positive patients discovered that concomitant DCIS was associated with smaller tumours and less node involvement.^7^ Similar to these studies, our study found that the DCIS component in IDC was more common in younger patients and was significantly correlated with lower IDC grade. However, no study has discussed the relationship between concomitant DCIS and the 21-gene RS. During the development and validation of the 21-gene assay, several clinicopathological characteristics were found to correlate with the 21-gene RS: younger age, higher tumour grade and lower PR expression were correlated with higher RS results.^10,17,18^ In addition to these previous findings, our study discovered that the DCIS component in IDC also impacted the 21-gene RS, as concomitant DCIS in IDC was independently related to lower 21-gene RS, which may be due to the relatively better biological behaviour in IDC patients with the DCIS component.

Since it has been acknowledged that DCIS has a different behaviour than IDC, some studies have focused on the proliferation- and invasion-related gene expression between DCIS and IDC tumours. When comparing pure DCIS samples with invasive breast carcinoma samples, Solin et al. found that the expression levels of the proliferation genes from the 21-gene panel were significantly lower in pure DCIS tumours.^19^ Toss et al. also found that IDC/DCIS patients had higher CTSL2 expression than pure DCIS patients.^20^ Furthermore, among IDC/DCIS patients, CTSL2 expression was higher in the invasive component than the DCIS component.^20^ Moreover, Gonzalez et al. assessed the MMP expression in IDC tumours with or without a DCIS component and discovered a higher expression of MMP11 in pure IDC tumours than in IDC/DCIS tumours.^21^ Consistent with those findings, when exploring the expression of the 16 cancer genes between IDC and IDC/DCIS tumours, we found that genes in the proliferation group (including Ki67, CCNB1 and MYBL2) and invasion group (MMP11 and CTSL2) in the 21-gene panel were significantly lower in IDC/DCIS tumours than in pure IDC tumours.

Since the DCIS component has been proven to impact the biological behaviour of tumours, it would be interesting to know whether the proportion of DCIS would further influence the tumour biology and the 21-gene RS. Wong et al. found that an increasing DCIS component correlated with a lower Ki67 expression level and less node involvement.^8^ In our study, we demonstrated that IDC/DCIS ≥ 50% patients had a significantly lower 21-gene RS than IDC/DCIS < 50% patients. Moreover, regarding the single gene expression levels among the 21-gene panel, we found that only MMP11 and CD68 remained independently different between these two groups. As a key element in tumour invasion and metastasis, a high level of MMP11 was previously proven to correlate with poor prognosis in invasive breast cancer patients.^22,23^ According to our study, the decrease in MMP11 expression in IDC/DCIS tumours may play an important role in having a relatively low RS, which might also lead to better tumour biological behaviour. The different expression of CD68, which is a marker of macrophages,^24^ between IDC and IDC/DCIS patients, might suggest a different TIL level between IDC and IDC/DCIS tumours, which warrants further investigation.

The effect of DCIS grade on invasive tumour biology is uncertain. A Korean cohort study of 1751 patients discovered that high DCIS grade was an independent prognostic factor in IDC/DCIS patients.^25^ A randomised European Organization for Research and Treatment of Cancer (EORTC) trial reported that DCIS grade was not correlated with the risk of invasive tumour recurrence.^26^ Interestingly, in our study, a lower DCIS grade was found to be independently associated with a lower 21-gene RS among IDC/DCIS patients, which might suggest that a low DCIS grade is related to favourable biology, even for invasive tumours.

It has not been well evaluated whether concurrent DCIS in IDC tumours would influence chemotherapy decision-making. Studies including all molecular subtypes reported that the DCIS component was correlated with less chemotherapy administration.^6,27^ However, in our study, no difference in chemotherapy usage was observed between IDC and IDC/DCIS patients. This may be because our patients had ER-positive, HER2-negative disease. Moreover, our patients were all tested with the 21-gene RS, which could have helped clinicians make more precise chemotherapy decisions and decrease the impact of DCIS components on chemotherapy usage.

The prognostic value of the DCIS component in IDC is still controversial. With 2239 ER-positive patients who underwent mastectomy without adjuvant radio- or chemotherapy, Dieterich et al.^7^ found that IDC with a DCIS component was significantly associated with lower local recurrence. However, Goh et al. analysed data from 3001 patients with all molecular subtypes and found that the improvement in DFS among IDC/DCIS patients was only observed in the HER2-positive group.^6^ Chagpar et al. reported that IDC with a DCIS component had favourable features but was not an independent factor in improving disease outcomes.^4^ Moreover, based on the Surveillance, Epidemiology and End Results (SEER) database, Wu et al. demonstrated that high DCIS (DCIS ≥ 25%) was associated with favourable BCSS compared to pure IDC, but the survival advantage disappeared after propensity score matching.^27^ In our study, no difference in DRFi was observed between IDC and IDC/DCIS patients. However, due to the relatively short follow-up time and small number of events, our results should be interpreted with caution. Furthermore, we analysed the prognostic value of the 21-gene RS in patients with and without a DCIS component and found that 21-gene RS significantly predicted DRFi for all patients and may predict DRFi for pure IDC patients with borderline significance, but was not associated with DRFi among IDC/DCIS patients. Notably, after the development of the 21-gene RS assay, Solin et al. generated a multigene assay to predict the risk of local recurrence among pure DCIS patients, which was called the DCIS score.^19,28^ In DCIS patients, the DCIS score was significantly correlated with local recurrence after breast-conserving therapy, while the 21-gene RS was not.^19^ This raised the question whether the 21-gene RS was accurate in predicting disease outcome in IDC patients with a high DCIS proportion. It also raised the question whether the 21-gene RS has the same cost-effectiveness in IDC/DCIS patients as among pure IDC patients, especially when the proportion of DCIS components is high.

The strength of our study was that we comprehensively analysed the 21-gene RS assay in IDC patients with or without a DCIS component. To our knowledge, this is the first study to explore the influence of DCIS components on the 21-gene RS. However, the current study still has some limitations. First, the study was single-centred and retrospectively designed, which could cause certain selection bias. Second, the follow-up time of our study was relatively short (31 months), and the number of events was relatively small, especially for ER-positive, HER2-negative patients, who have a long-term risk of disease recurrence. Moreover, the event rate of distant recurrence was rather low, particularly in IDC/DCIS patients (four distant recurrence events), which limits the power of our study to address the prognostic value of the 21-gene RS in IDC patients with concurrent DCIS. Therefore, interpretation of the prognostic results of our study needs caution. Finally, in our study, the 21-gene RS was conducted after macrodissection rather than microdissection, since the majority of IDC/DCIS tumours had rather mixed IDC and DCIS components that were hard to separate, which limited our ability to compare gene expression between the pure IDC and IDC parts of IDC/DCIS tumours. We believe that such an analysis is warranted in the future to investigate the behaviour of IDC derived from DCIS and de novo IDC.

## Conclusion

The DCIS component in IDC was associated with a lower 21-gene RS, which may be correlated with lower expression levels of proliferation and invasion genes. Among IDC patients with concomitant DCIS, a higher DCIS proportion and lower DCIS grade were independently correlated with a lower 21-gene RS. The DCIS component in IDC tumours did not influence chemotherapy usage or prognosis, which deserves further clinical evaluation.

## Supplementary information

Supplementary information

## Data Availability

The datasets used and analysed during this study are available from the corresponding author on reasonable request.
