# Supplementary material for: Comprehensive analysis of the 21-gene recurrence score in invasive ductal breast carcinoma with or without ductal carcinoma in situ component
Source: Br J Cancer. 2020 Dec 17;124(5):975–81. doi: 10.1038/s41416-020-01212-w (PMC7921681; doi:10.1038/s41416-020-01212-w)
Supplement: Supplementary file 1 — Supplementary information [file 41416_2020_1212_MOESM1_ESM.docx]

**Supplementary Table 1.** Association of 21-gene RS and characteristics among IDC/DCIS patients

| Characteristics | Total, No. | 21-gene RS | | | P |
| --- | --- | --- | --- | --- | --- |
|  |  | Low, No.(%) | Intermediate, No.(%) | High, No.(%) |  |
| Total | 320 | 74 (23.1%) | 182 (56.9%) | 64 (20.0%) |  |
| Age (years) | |  |  |  | 0.395 |
| <50 | 134 | 32 (23.9%) | 80 (59.7%) | 22 (16.4%) |  |
| ≥50 | 186 | 42 (22.6%) | 102 (54.8%) | 42 (22.6%) |  |
| Tumor stage | |  |  |  | 0.394 |
| T1 | 221 | 54 (24.4%) | 127 (57.5%) | 40 (18.1%) |  |
| T2-3 | 99 | 20 (20.2%) | 55 (55.6%) | 24 (24.2%) |  |
| Nodal status | |  |  |  | 0.745 |
| pN0 | 266 | 62 (23.3%) | 149 (56.0%) | 55 (20.7%) |  |
| pN1 | 54 | 12 (22.2%) | 33 (61.1%) | 9 (16.7%) |  |
| DCIS proportion | |  |  |  | 0.057 |
| <50% | 206 | 39 (18.9%) | 123 (59.7%) | 44 (21.4%) |  |
| ≥50% | 114 | 35 (30.7%) | 59 (51.8%) | 20 (17.5%) |  |
| DCIS grade | |  |  |  | **<0.001** |
| I | 23 | 8 (34.8%) | 11 (47.8%) | 4 (17.4%) |  |
| II | 194 | 55 (28.4%) | 113 (58.2%) | 26 (13.4%) |  |
| III | 103 | 11 (10.7%) | 58 (56.3%) | 34 (33.0%) |  |
| Molecular subtype | |  |  |  | **<0.001** |
| Luminal A | 122 | 42 (34.4%) | 68 (55.7%) | 12 (9.8%) |  |
| Luminal B | 198 | 32 (16.2%) | 114 (57.6%) | 52 (26.3%) |  |

*IDC grade unknown in 4 patients. Missing values are missing at complete random, therefore pairwise deletion was performed when conducting chi-square test.

Abbreviation: IDC = invasive ductal carcinoma; DCIS = ductal carcinoma in situ; RS = recurrence score.

**Supplementary Table 2.** Association of patient characteristics and use of adjuvant chemotherapy

| Characteristics | Adjuvant chemotherapy | | P |
| --- | --- | --- | --- |
|  | No, No. (%) | Yes, No. (%) |  |
| Total | 636 (43.6%) | 822 (56.4%) |  |
| Age (years) |  |  | **<0.001** |
| <50 | 171 (35.6%) | 309 (64.4%) |  |
| ≥50 | 465 (47.5%) | 513 (52.5%) |  |
| Surgery type |  |  | 0.192 |
| Mastectomy | 324 (42.0%) | 447 (58.0%) |  |
| Lumpectomy | 312 (45.4%) | 375 (54.6%) |  |
| Tumor stage |  |  | **<0.001** |
| T1 | 505 (48.6%) | 534 (51.4%) |  |
| T2-3 | 128 (30.9%) | 286 (69.1%) |  |
| Nodal status |  |  | **<0.001** |
| pN0 | 602 (49.2%) | 622 (50.8%) |  |
| pN1 | 34 (14.5%) | 200 (85.5%) |  |
| Histological type |  |  | 0.113 |
| Pure IDC | 484 (42.5%) | 654 (57.5%) |  |
| IDC/DCIS | 152 (47.5%) | 168 (52.5%) |  |
| IDC grade |  |  | **<0.001** |
| I | 104 (73.8%) | 37 (26.2%) |  |
| II | 459 (47.6%) | 506 (52.4%) |  |
| III | 68 (20.2%) | 268 (79.8%) |  |
| Molecular subtype |  |  | **<0.001** |
| Luminal A | 312 (69.6%) | 136 (30.4%) |  |
| Luminal B | 324 (32.1%) | 686 (67.9%) |  |
| ER expression |  |  | **0.001** |
| <50% | 29 (28.2%) | 74 (71.8%) |  |
| ≥50% | 607 (44.8%) | 748 (55.2%) |  |
| PR expression |  |  | **<0.001** |
| <20% | 123 (27.2%) | 329 (72.8%) |  |
| ≥20% | 513 (51.0%) | 493 (49.0%) |  |
| Ki67 expression |  |  | **<0.001** |
| <14% | 394 (60.9%) | 253 (39.1%) |  |
| ≥14% | 241 (29.8%) | 569 (70.2%) |  |
| RS |  |  | **<0.001** |
| <18 | 216 (74.2%) | 75 (25.8%) |  |
| 18-30 | 243 (44.9%) | 420 (55.1%) |  |
| ≥31 | 78 (19.3%) | 327 (80.7%) |  |

Abbreviation: IDC = invasive ductal carcinoma; DCIS = ductal carcinoma in situ; ER = estrogen receptor; PR = progesterone receptor; RS = recurrence score.

**Supplementary Table 3.** Multivariate analysis of patient characteristics and use of adjuvant chemotherapy

| Characteristics | OR* | 95%CI | P |
| --- | --- | --- | --- |
| Age <50 vs. ≥50 | 2.83 | 2.11-3.80 | **<0.001** |
| T2-3 vs. T1 | 1.50 | 1.11--2.03 | **0.009** |
| N1 vs. N0 | 10.64 | 6.71-16.87 | **<0.001** |
| IDC Grade |  |  | **<0.001** |
| II vs. I | 1.82 | 1.14-2.92 | **0.012** |
| III vs. I | 3.81 | 2.16-6.73 | **<0.001** |
| Luminal A vs. B | 1.59 | 0.88-2.85 | 0.124 |
| ER <50% vs. ≥50% | 0.96 | 0.54-1.70 | 0.890 |
| PR <20% vs. ≥20% | 2.55 | 1.64-3.95 | **<0.001** |
| Ki67 <14% vs. ≥14% | 0.42 | 0.25-0.70 | **0.001** |
| RS |  |  | **<0.001** |
| Intermediate vs. Low | 3.49 | 2.45-4.96 | **<0.001** |
| High vs. Low | 10.99 | 7.18-16.81 | **<0.001** |

*Odds ratio indicated the likelihood of receiving adjuvant chemotherapy compared to not receiving adjuvant chemotherapy.

Abbreviation: OR = odds ratio; CI = confidence interval; IDC = invasive ductal carcinoma; ER = estrogen receptor; PR = progesterone receptor; RS = recurrence score.

**Supplementary Table 4**. Chemotherapy rate among patients with different recurrence score

| **RS** | **IDC** | **IDC/DCIS** | **P** |
| --- | --- | --- | --- |
| Low-risk | 63/217 (29.0%) | 12/74 (16.2%) | **0.030** |
| Intermediate-risk | 318/580 (54.8%) | 102/183 (56.0%) | 0.773 |
| High-risk | 273/341 (80.1%) | 54/64 (84.4%) | 0.422 |

Abbreviation: IDC = invasive ductal carcinoma; DCIS = ductal carcinoma in situ; RS = recurrence score.

**Supplementary Table 5.** Detailed events of patients with different RS and histopathologic type

| RS | Group total events  No. (%) | LRR | CBC | distant recurrence | Second primary cancer | death | |
| --- | --- | --- | --- | --- | --- | --- | --- |
|  |  |  |  |  |  | breast | other cause |
| IDC |  |  |  |  |  |  |  |
| Low | 10/217 (4.6%) | 0 | 2 | 4 | 3 | 0 | 1 |
| intermediate | 24/580 (4.1%) | 3 | 4 | 4 | 8 | 1 | 4 |
| high | 31/341 (9.1%) | 7 | 5 | 6 | 4 | 6 | 3 |
| IDC/DCIS |  |  |  |  |  |  |  |
| Low | 1/74 (1.4%) | 1 | 0 | 0 | 0 | 0 | 0 |
| intermediate | 6/183 (3.3%) | 0 | 2 | 2 | 2 | 0 | 0 |
| high | 5/64 (7.8%) | 2 | 0 | 2 | 0 | 0 | 1 |

Abbreviation: IDC = invasive ductal carcinoma; DCIS = ductal carcinoma in situ; RS = recurrence score; LRR = locoregional recurrence; CBC = contralateral breast cancer.

**Supplementary Table 6.** Prognosis in patients with different RS and different histological type.

| RS | DRFi event  No. (%) | P^a^ | DFS event  No. (%) | P^a^ | OS event  No. (%) | P^a^ |
| --- | --- | --- | --- | --- | --- | --- |
| All patients |  |  |  |  |  |  |
| Low risk | 4 (1.4) | **0.010** | 11 (3.8) | **0.001** | 1 (0.3) | **0.012** |
| Intermediate risk | 7 (0.9) |  | 30 (3.9) |  | 5 (0.7) |  |
| High risk | 14 (3.5) |  | 36 (8.9) |  | 10 (2.5) |  |
| IDC |  |  |  |  |  |  |
| Low risk | 4 (1.8) | **0.018** | 10 (4.6) | **0.006** | 1 (0.5) | **0.044** |
| Intermediate risk | 5 (0.9) |  | 24 (4.1) |  | 5 (0.9) |  |
| High risk | 12 (3.5) |  | 31 (9.1) |  | 9 (2.6) |  |
| IDC/DCIS |  |  |  |  |  |  |
| Low risk | 0 (0.0) | 0.293 | 1 (1.4) | 0.141 | 0 (0.0) | 0.200 |
| Intermediate risk | 2 (1.1) |  | 6 (3.3) |  | 0 (0.0) |  |
| High risk | 2 (3.1) |  | 5 (3.8) |  | 1 (1.6) |  |

Abbreviation: IDC = invasive ductal carcinoma; DCIS = ductal carcinoma in situ; RS = recurrence score; DRFi = distant recurrence free interval; DFS = distant recurrence survival; OS = overall survival.

^a^ chi-square test or Fisher exact test as appropriate.

**Supplementary Table 7.** Univariate Cox regression analysis of factors associated with DRFi in pure IDC patients and IDC/DCIS patients

| Characteristics | IDC | | |  | IDC/DCIS | | |
| --- | --- | --- | --- | --- | --- | --- | --- |
|  | HR | 95%CI | P |  | HR | 95%CI | P |
| Age ≥50 vs. <50 | 0.92 | 0.37-2.29 | 0.861 |  | 0.77 | 0.11-5.50 | 0.798 |
| T2-3 vs. T1 | 3.58 | 1.51-8.51 | **0.004** |  | 2.26 | 0.32-16.06 | 0.416 |
| N1 vs. N0 | 1.92 | 0.63-5.85 | 0.251 |  | 1.86 | 0.19-17.93 | 0.591 |
| Luminal B vs. A | 8.49 | 1.14-63.26 | **0.037** |  | 46.44 | 0.01-∞ | 0.363 |
| RS |  |  | 0.099 |  |  |  | 0.704 |
| Intermediate vs. Low | 0.61 | 0.16-2.27 | 0.458 |  | ∞ | 0.00-∞ | 0.953 |
| High vs. Low | 1.85 | 0.60-5.74 | 0.286 |  | ∞ | 0.00-∞ | 0.950 |
| DCIS grade |  |  | N/A |  |  |  | 0.554 |
| II vs. I | N/A | N/A | N/A |  | 1.00 | 0.00-∞ | 1.000 |
| III vs. I | N/A | N/A | N/A |  | ∞ | 0.00-∞ | 0.735 |
| DCIS proportion ≥50% vs. <50% | N/A | N/A | N/A |  | 1.49 | 0.21-10.59 | 0.692 |

Abbreviation: HR = hazard ratio; CI = confidence interval; DRFi = distant recurrence free interval; IDC = invasive ductal carcinoma; DCIS = ductal carcinoma in situ; RS = recurrence score.

**Supplementary Table 8.** Multivariate Cox regression analysis of factors associated with DRFi in pure IDC patients

| Characteristics | HR | 95%CI | P |
| --- | --- | --- | --- |
| T2-3 vs. T1 | 3.17 | 1.33-7.54 | **0.009** |
| Luminal B vs. A | 6.75 | 0.90-50.68 | 0.063 |
| RS |  |  | 0.185 |
| Intermediate vs. Low | 0.55 | 0.15-2.05 | 0.371 |
| High vs. Low | 1.46 | 0.47-4.53 | 0.517 |

Abbreviation: HR = hazard ratio; CI = confidence interval; DRFi = distant recurrence free interval; IDC = invasive ductal carcinoma; RS = recurrence score.


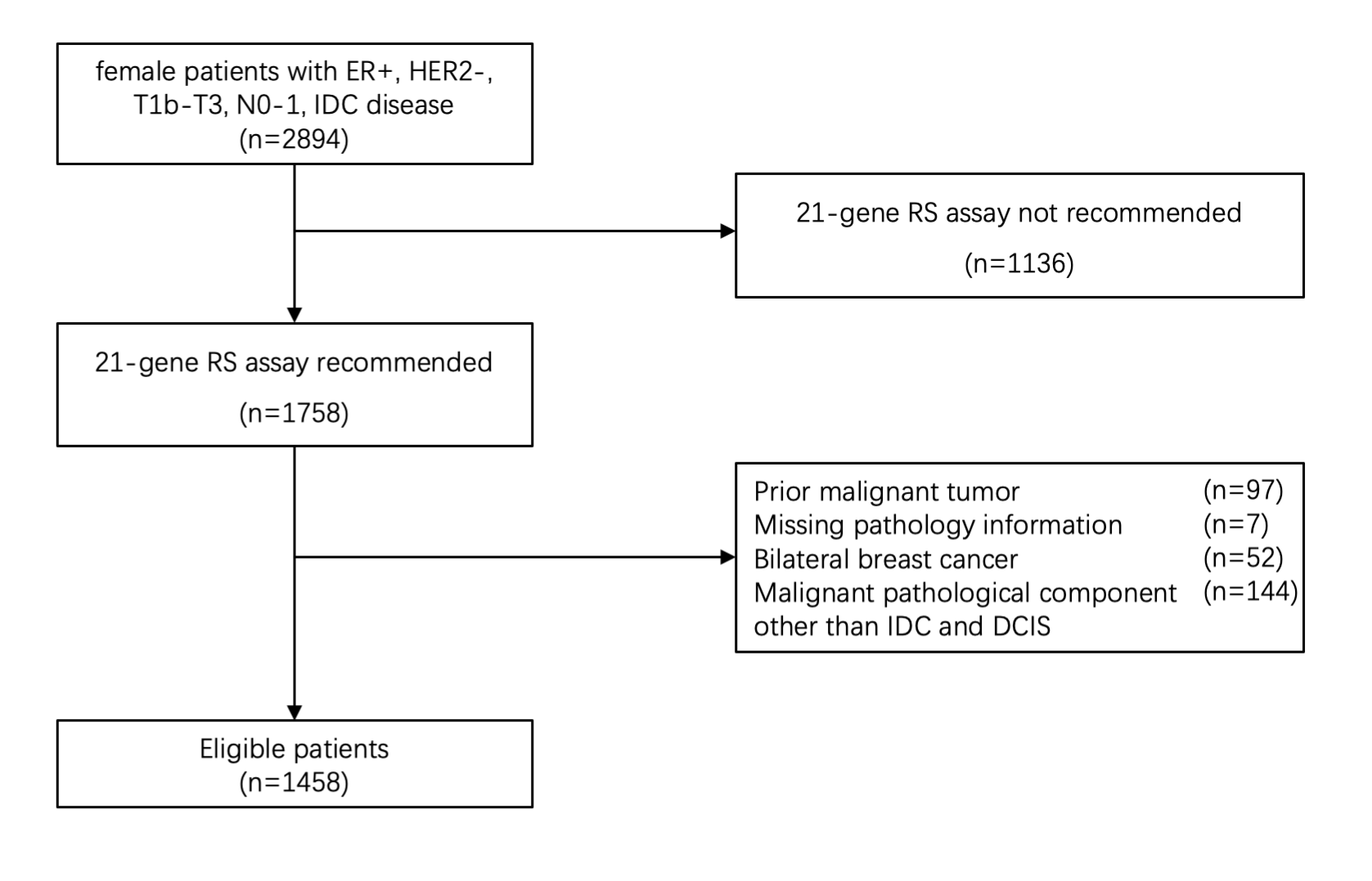


**Supplementary Figure 1.** Study flowchart

Abbreviation: ER = estrogen receptor; HER2 = human epidermal growth factor receptor 2; RS = recurrence score; IDC = invasive ductal carcinoma; DCIS = ductal carcinoma in situ.

**
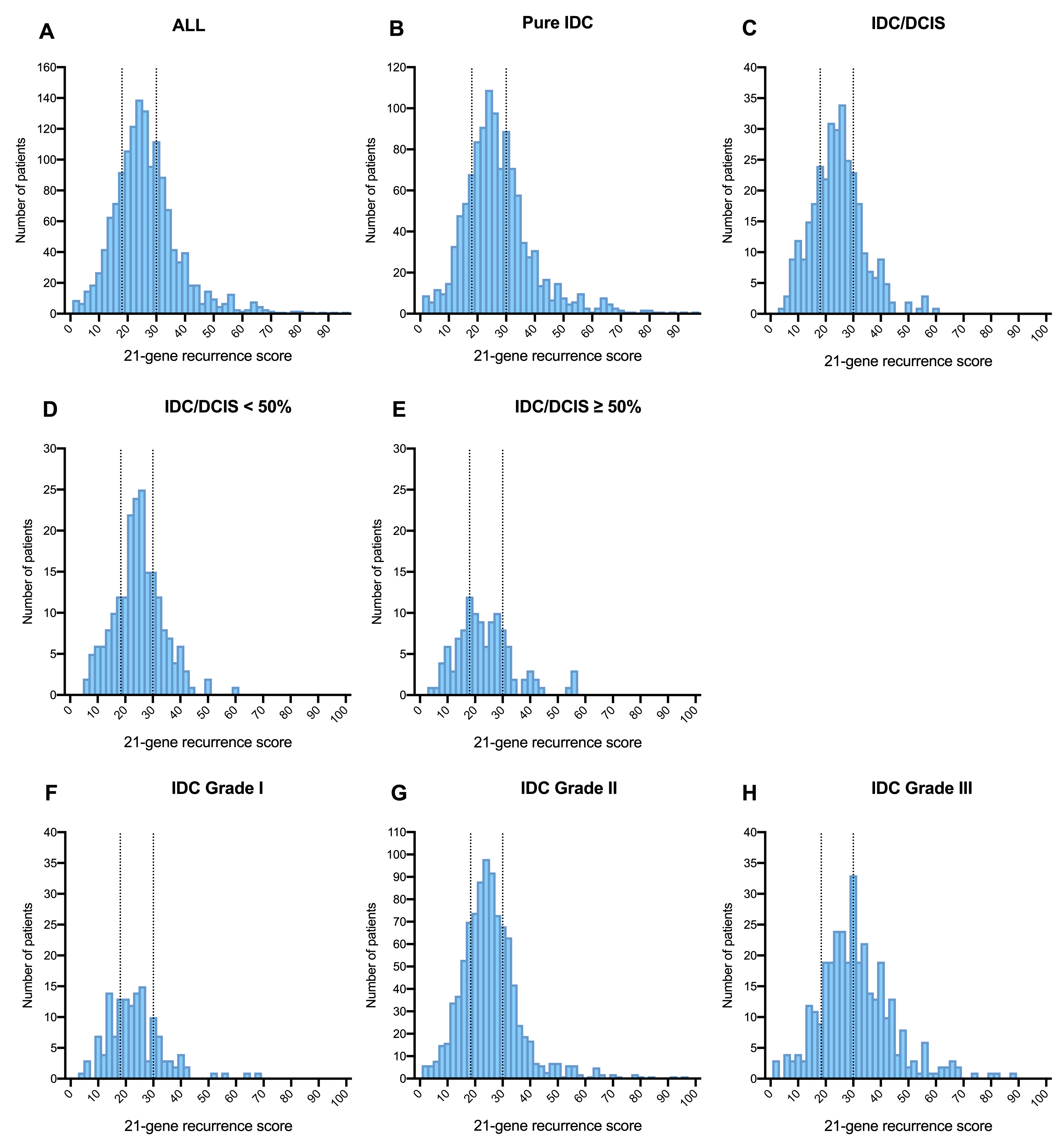
**

**Supplementary Figure 2.** Histogram of the distribution of 21-gene recurrence score in A) all patients; B) pure IDC patients; C) IDC/DCIS patients; D) IDC/DCIS<50% patients; E) IDC/DCIS≥50% patients; F) IDC grade I patients; G) IDC grade II patients; H); IDC grade III patients.

Abbreviation: IDC = invasive ductal carcinoma; DCIS = ductal carcinoma in situ


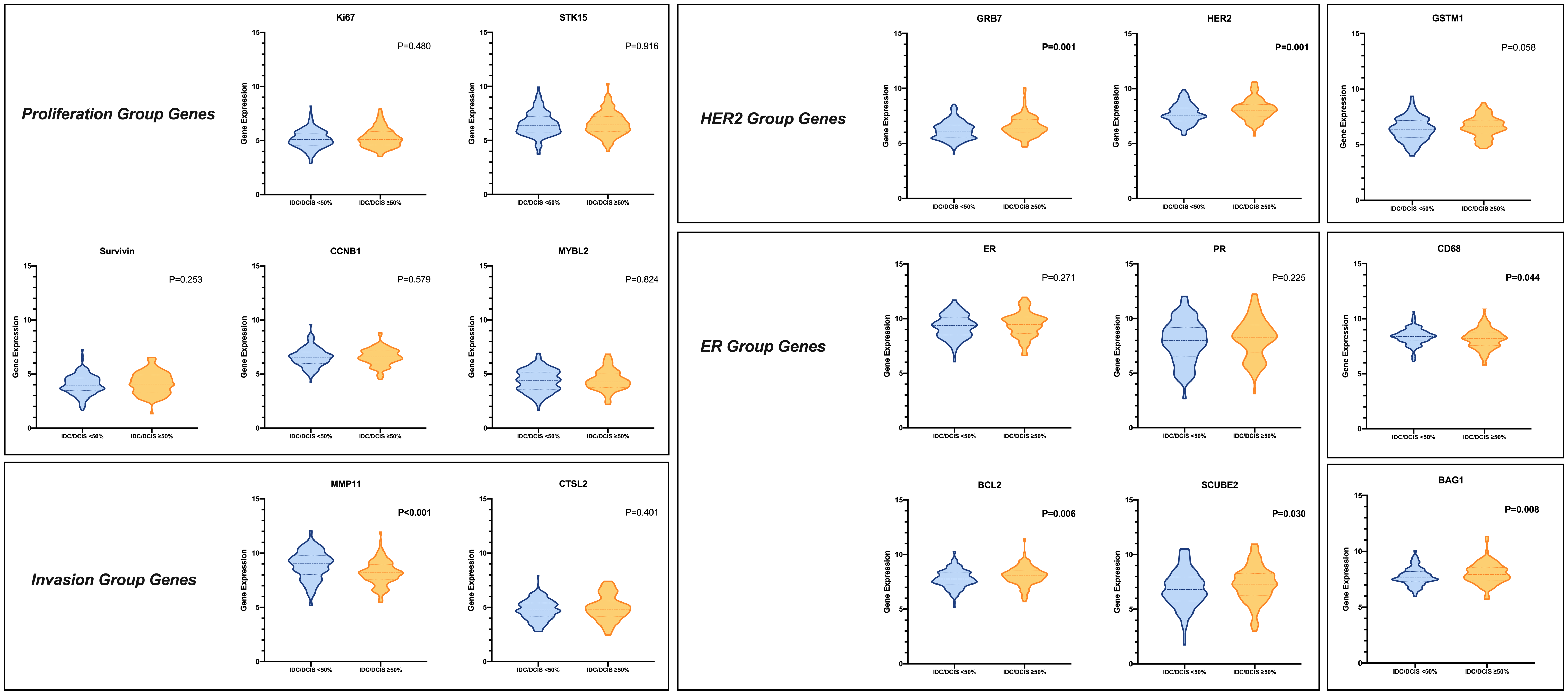


**Supplementary Figure 3.** Individual gene expression level of the 16 cancer genes from 21-gene recurrence score in breast cancer patients with different proportion of DCIS.

Abbreviation: DCIS = ductal carcinoma in situ.


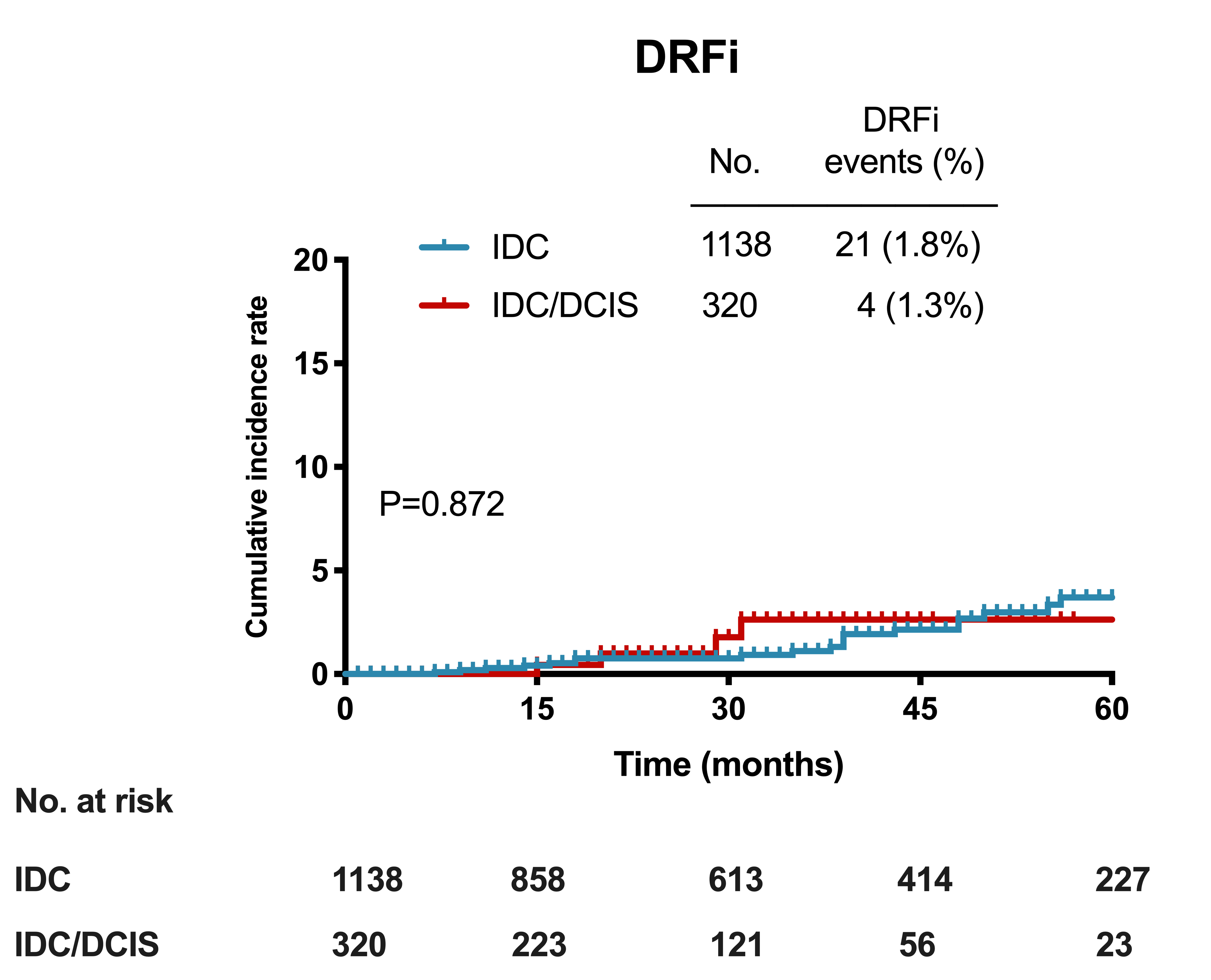


**Supplementary Figure 4.** Kaplan-Meier curve of cumulative incidence rate of DRFi stratified by histopathological type.

Abbreviation: DRFi = distant recurrence free interval; IDC = invasive ductal carcinoma; DCIS = ductal carcinoma in situ.


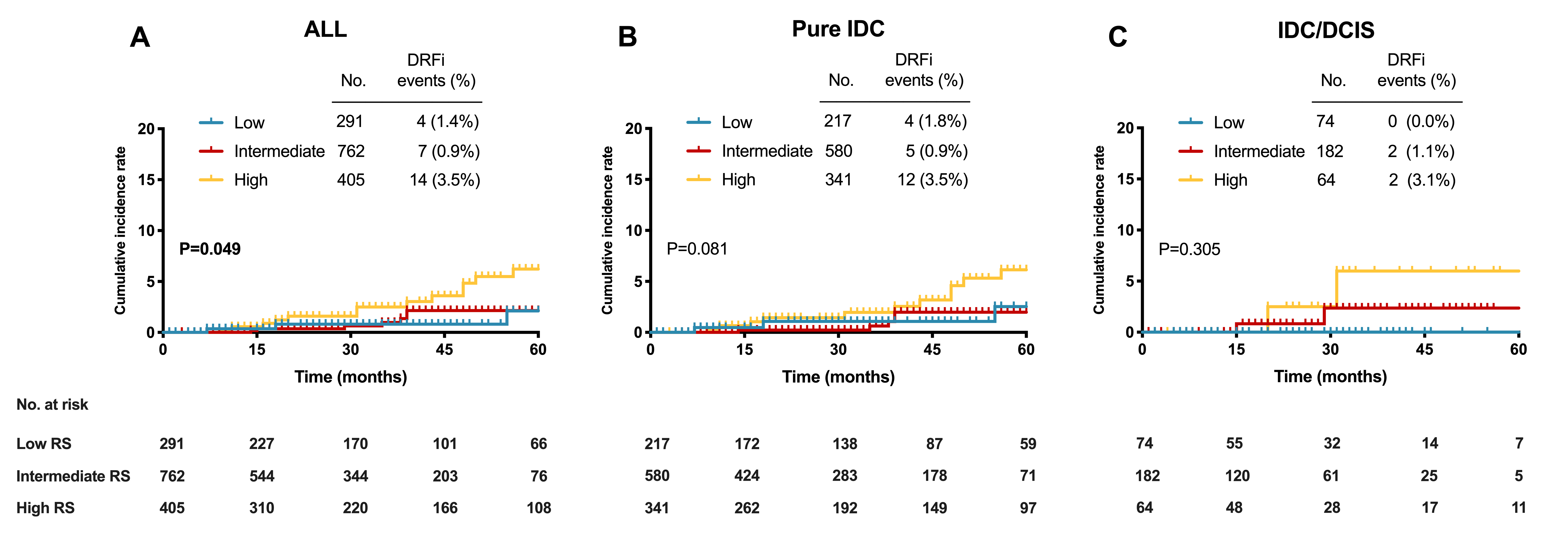


**Supplementary Figure 5.** Kaplan-Meier curve of cumulative incidence rate of DRFi stratified by 21-gene RS A) in whole patient cohort; B) in pure IDC patients; C) in IDC/DCIS patients.

Abbreviation: DRFi = distant recurrence free interval; IDC = invasive ductal carcinoma; DCIS = ductal carcinoma in situ.

**
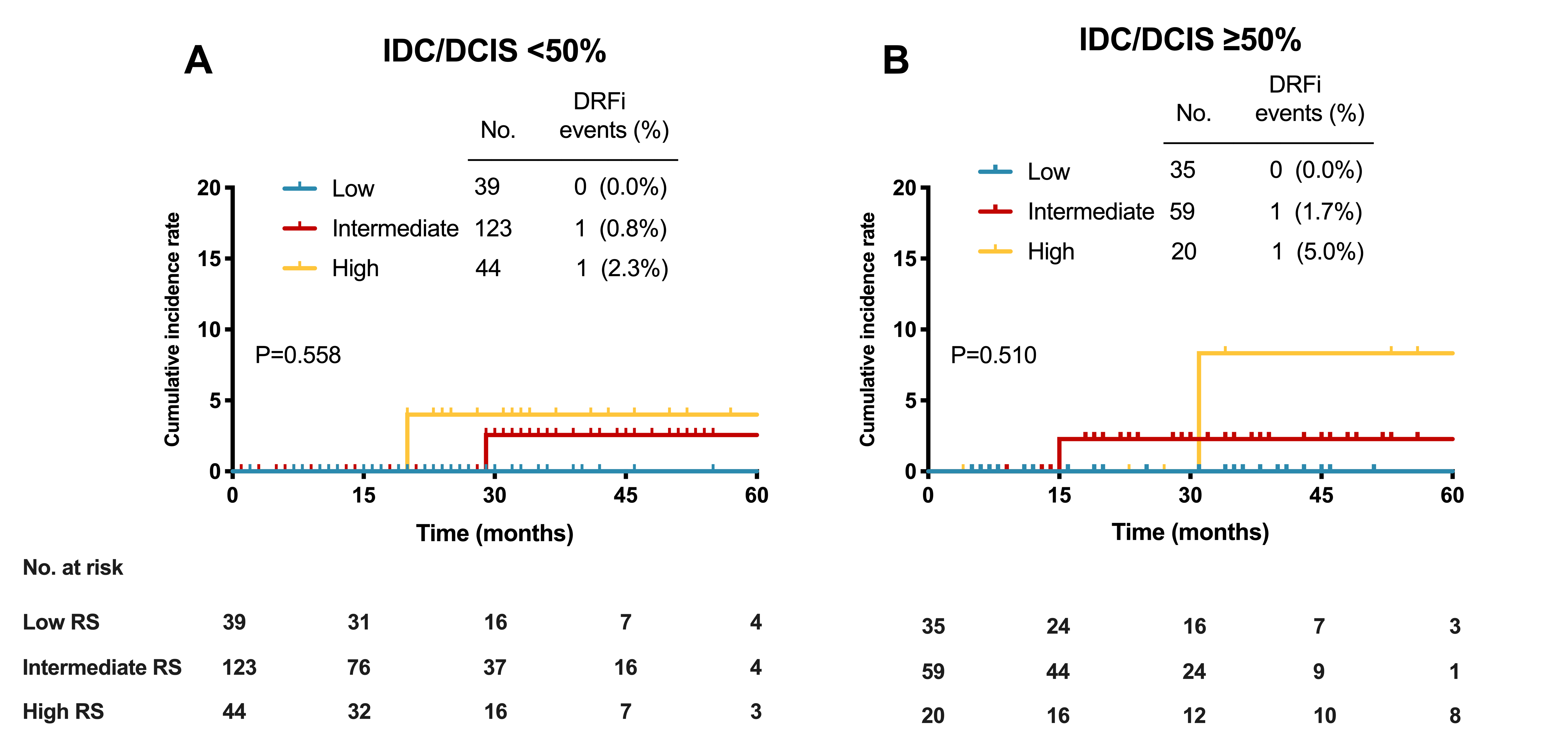
**

**Supplementary Figure 6.** Kaplan-Meier curve of cumulative incidence rate of DRFi stratified by 21-gene RS A) in IDC/DCIS<50% patients; B) in IDC/DCIS≥50% patients.

Abbreviation: DRFi = distant recurrence free interval; IDC = invasive ductal carcinoma; DCIS = ductal carcinoma in situ.
